# eHealth Literacy and Health-Related Internet Use Among Swedish Primary Health Care Visitors: Cross-Sectional Questionnaire Study

**DOI:** 10.2196/63288

**Published:** 2024-12-05

**Authors:** Anna Sjöström, Senada Hajdarevic, Åsa Hörnsten, Ulf Isaksson

**Affiliations:** 1 Department of Nursing, Umea University, Biology Building, Umeå, 90187, Sweden, 46 0702353499

**Keywords:** eHealth literacy, primary health care, health-related internet information, health literacy, digitalization, eHealth, cost-effective care, internet, health applications, digital health, patient participation, health behaviors, questionnaire, well-being

## Abstract

**Background:**

Digitalization has profoundly transformed health care delivery, especially within primary health care, as a crucial avenue for providing accessible, cost-effective care. While eHealth services are frequently highlighted for improving health care availability and promoting equality, it is essential to recognize that digitalization can inadvertently exclude individuals who lack the prerequisites to use eHealth services, that is, those with low eHealth literacy. Previous research has identified lower eHealth literacy among older individuals, those with lower educational levels, and those who use the internet less frequently. However, in a Swedish context, only a few studies have investigated eHealth literacy.

**Objective:**

This study investigated eHealth literacy and its association with health-related internet use and sociodemographic characteristics among primary health care visitors.

**Methods:**

This cross-sectional study used a quantitative, descriptive approach. Swedish-speaking patients visiting a primary health care center participated by answering the multidimensional eHealth Literacy Questionnaire (eHLQ) and questions regarding sociodemographic characteristics and internet usage. The study compared mean scores using the Mann-Whitney *U* test and the Kruskal-Wallis test. A logistic regression analysis also explored the associations between eHealth literacy and significant independent variables identified in the univariate analyses.

**Results:**

As a group, the 172 participants rated highest in understanding and engagement with their health (median eHLQ score 3, IQR 2.8‐3.4), as well as in feeling secure about the confidentiality of eHealth services (median eHLQ score 3, IQR 2‐3), while they rated lower in motivation to use eHealth (median eHLQ score 2.6, IQR 2‐3), the suitability of eHealth services to their personal needs (median eHLQ score 2.75, IQR 2‐3), and their perceived ability to understand and use health-related internet information (median eHLQ score 2.6, IQR 2‐3). The logistic regression analysis identified that lower eHealth literacy was associated with older age, particularly in domains related to finding, understanding, and using health-related internet information (odds ratio [OR] 1.02, 95% CI 1‐1.05; *P*=.03); digital technology use (OR 1.05, 95% CI 1.02‐1.08; *P*<.001); and accessing well-functioning eHealth services (OR 1.02, 95% CI 1‐1.05; *P*=.03). Additionally, in the logistic regression analysis, perceiving health-related internet information as not useful was linked to lower literacy in all eHLQ domains except one.

**Conclusions:**

Our findings regarding the primary challenges within our sample underscore the importance of developing and tailoring eHealth services to accommodate users’ individual needs better, enhancing motivation for eHealth use, and continuing efforts to improve overall health literacy. These measures, which both eHealth developers and health care professionals should consider, are crucial for addressing the digital divide and expanding access to eHealth services for as many people as possible.

## Introduction

Digitalization has significantly transformed the delivery of health care. Particularly within primary health care, which faces challenges related to an increasingly aging and multimorbid population, eHealth is an important means for providing accessible and cost-effective care [1,2]. eHealth refers to information and communications technologies used in health care, including health-related internet information (HRII), electronic health records and prescriptions, health applications, and digital health care visits [3].

In a Swedish context, the government envisions that by 2025, Sweden will be the world leader in using eHealth to facilitate equitable access to quality health care and enhance patient participation. This visionary perspective underscores the significance of developing eHealth services based on citizens’ needs and prerequisites [4]. Recent reports from the Swedish Internet Foundation have indicated that 96% of all adult Swedish citizens use the internet, with 83% using eHealth services, including HRII acquisition, digital health care visits, and health application use [5,6]. Similar to global trends, the use of eHealth services in Sweden experienced a substantial increase during the COVID-19 pandemic, with digital alternatives largely replacing traditional health care practices due to social distancing measures [7].

The internet is also a common source of health information; for many individuals, it is the primary choice [5,8]. The advantages of reliable HRII include its potential to enhance people’s self-management capabilities, increase involvement in care, and improve the ability to make informed decisions. These, in turn, facilitate person-centered communication in health care [9,10]. In a previous study, Swedish primary health care nurses emphasized similar advantages with credible HRII, exemplified by the digital national health care platform 1177.se, where residents can access quality-reviewed general health information, self-care advice, and personal health data [11,12]. However, these primary health care nurses also highlighted challenges associated with HRII, including patient anxiety and confusion. They noted conflicted consultations arising from patients encountering unreliable HRII and experiencing difficulties interpreting this information [12]. Many individuals initiate health information searches on Google, a practice frequently highlighted in the literature as potentially problematic due to the risk of directing users to unreliable websites [12-15].

While eHealth services are often emphasized for enhancing health care availability and promoting health care equality, digitalization can exclude individuals who lack the prerequisites to use such services [16]. Increased health inequities due to digitalization are recognized as the “digital divide” [16,17]. Digital alienation is associated with risk factors such as advanced age, low educational attainment, and physical limitations [17,18]. Other contributing factors include lack of motivation, limited digital access, low self-confidence, and inadequate knowledge and abilities [18].

In 2006, Norman and Skinner [19] initially described eHealth literacy as the ability to search, find, appraise, and use health information from digital sources. However, with the evolution of digital technologies and the integration of more interactive web-based elements, such as social media, the concept requires redefinition [20]. As part of this effort, Norgaard et al [21] adopted a validity-driven approach to incorporate elements related to the interaction between individuals and eHealth systems, leading to the development of the 7-domain eHealth Literacy Framework (eHLF). The 7 domains in the eHLF encompass both individual capabilities and characteristics of eHealth services, emphasizing their interaction [21]. Based on the eHLF, the eHealth Literacy Questionnaire (eHLQ) was developed to assess individuals’ eHealth literacy across multiple domains [22].

Adequate eHealth literacy has been associated with positive health behaviors, such as increased exercise, balanced nutrition, and improved stress management among various patient groups [23,24]. Moreover, individuals with adequate eHealth literacy are inclined to conduct more frequent HRII searches. This behavior contributes to enhanced health knowledge and yields positive outcomes, including a better understanding of medical conditions, increased empowerment, improved self-management capacity, enhanced communication with health care professionals, and greater engagement in medical decision-making [25].

Previous research has consistently identified lower eHealth literacy among older individuals, those with lower levels of education, and individuals who use the internet less frequently [26-28]. However, in the Swedish context, only a limited number of studies have investigated eHealth literacy, primarily focusing on specific demographic groups such as parents, Arabic-speaking immigrants, and older populations [29-31]. Notably, there is a lack of Swedish studies examining eHealth literacy among primary health care visitors from a multidimensional perspective.

To maximize the positive impact of eHealth services, promote overall well-being, ensure health care accessibility, facilitate patient involvement, and alleviate strain on primary health care, it is essential for primary health care visitors to possess sufficient eHealth literacy [1,4,25]. It is essential to understand the specific challenges individuals face in order to effectively support them in improving their eHealth literacy. There is a knowledge gap regarding eHealth literacy among Swedish primary health care visitors, and the most challenging aspects of digital usage for this group remain unexplored. Therefore, this study aimed to investigate eHealth literacy from a multidimensional perspective, and its association with health-related internet use and sociodemographic characteristics among primary health care visitors.

## Methods

### Setting

Västerbotten County, with a population of approximately 137,000, exemplifies the structure of primary health care in Sweden, which shares similarities with primary health care center systems in other high-income nations, particularly serving as the initial point of contact in health care. A key distinction, however, is the extensive public management and tax-based funding of Swedish primary health care services [32]. The multidisciplinary teams in Swedish primary health care centers comprise physicians, specialist nurses, midwives, registered nurses, assistant nurses, physiotherapists, occupational therapists, psychologists, social workers, and dietitians. Common reasons for primary health care center visits include infections, pain disorders, respiratory issues, and mental health concerns, with patients frequently attending for the management of chronic conditions such as hypertension, type 2 diabetes, and asthma [33].

### Sample and Procedures

Data were collected from 2 rural and 2 urban primary health care centers of varying sizes (with patient lists ranging from 2500 to 20,000) in northern Sweden. Receptionists were tasked with distributing questionnaires to all adult (≥18 years old) Swedish-speaking patients visiting these health care centers within 2 weeks in November 2020. Participants could complete the questionnaire either at the primary health care center or at home, returning it by mail using a provided stamped and addressed envelope.

### Instrument and Data Collection

The eHLQ is based on the 7-domain eHLF [21,22]. This instrument consists of 35 items distributed across the 7 domains, categorized into 3 dimensions: individual competencies of the user, the user’s experiences with digital services, and the interaction between the user and digital services [22] (Table 1).

Each domain of the eHLQ comprises 4-6 items rated on a 4-point Likert scale, ranging from “strongly disagree” to “strongly agree.” The scoring for each item ranges from 1 to 4, with scores calculated as an index by averaging the item scores within each domain using equal weighting. The domains are presented separately, and no overall eHLQ score is calculated. Higher scores within each domain indicate stronger abilities or agreements with the domain’s focus [22].

The eHLQ was initially developed simultaneously in Danish and English, demonstrating psychometric solid properties in various contexts [22,34,35]. A systematic and rigorous process of translation and cultural adaptation was used for the Swedish translation, resulting in the validation of solid psychometric properties specific to the Swedish version [36]. In addition to the eHLQ, 2 additional items were included to assess participants’ perceptions of the usefulness and importance of HRII, using a 5-point Likert scale. Participants also self-assessed their health status on a 5-point scale ranging from poor to excellent. Sociodemographic information, including age, sex, education level, living arrangement, and work status, was collected. Furthermore, participants were asked about chronic diseases (see Table 2).

**Table 1.** Presentation of the eHLQ^a^ dimensions, domains, and the number of items in the respective domain.

eHLQ domain and dimension		Items, n
**Individual competence of the user**		
	1. Using technology to process health information	5
	2. Understanding of health concepts and language	5
I**nteraction between the user and the digital services**		
	3. Ability to actively engage with digital services	5
	4. Feeling safe and in control	5
	5. Motivated to engage with digital services	5
**The user´s experiences with digital services**		
	6. Access to digital services that work	6
	7. Digital services that suit individual needs	4

a eHLQ: eHealth Literacy Questionnaire.

**Table 2.** Sociodemographic characteristics of participants (N=172).

Characteristics			Values
**Sex, n (%)**			
	Male		77 (45.3)
	Female		93 (54.7)
	Not disclosed		2 (1.2)
**Age (years)**			
	Mean (SD)		57.5 (19.9)
	**n (%)**		
		≤40	44 (25.6)
		41‐60	41 (23.8)
		61‐74	43 (25)
		≥75	42 (24.4)
		Not disclosed	2 (1.2)
**Education, n (%)**			
	Elementary school or less		26 (15.1)
	Secondary school or vocational		75 (43.6)
	University		71 (41.3)
**Employment status, n (%)**			
	Working		70 (40.7)
	Unemployed		2 (1.2)
	Student		19 (11)
	Retired		76 (44.2)
	Other activity		5 (2.9)
**Living arrangement, n (%)**			
	Living with a partner or family		123 (71.3)
	Living alone		49 (28.7)
**Chronic disease, n (%)**			
	None		75 (44.6)
	Diabetes		29 (17.3)
	Cardiovascular disease		30 (17.9)
	Other chronic disease		34 (20.2)
**Self-rated health, n (%)**			
	Excellent or very good		51 (29.7)
	Good		64 (37.2)
	Somewhat okay or poor		55 (32)
	Not disclosed		2 (1.1)

### Data Analysis

Data analysis was performed using SPSS (version 25; IBM Corp) and JAMOVI (version 2.2.3; GitHub, Inc). Two questionnaires with ≥50% missing values were excluded. The expectation-maximization algorithm imputation in SPSS replaced internal missing values.

Demographic characteristics were presented using frequency and percentage for categorical variables, mean and SD with 95% CIs for continuous variables, and median and IQR as appropriate for skewed distributions. A box plot was used to visually illustrate data distribution across the 7 eHLQ domains. Participants were categorized into 4 age groups based on statistical considerations, representing quartiles of the total sample and guided by the theoretical rationale to enable comprehensive analyses of internet usage and eHealth literacy distribution.

Nonparametric tests were used due to deviations from normal distribution, as determined by the Shapiro-Wilk test and visual examination of the eHLQ and internet habit data. Mean eHLQ scores were compared using the Mann-Whitney *U* test, with effect size reported using Cohen *d* [37]. Cohen *d* values of 0.2, 0.5, and 0.8 represent small, medium, and large effect sizes, respectively. Comparisons involving more than 2 groups were analyzed using the Kruskal-Wallis test, and the effect size was assessed using epsilon squared. Epsilon squared values between 0 and 0.01 are considered negligible, 0.01 to 0.04 weak, >0.04 to 0.16 moderate, >0.16 to 0.36 relatively strong, and 0.36 to 0.64 strong [38].

Prior to conducting the multivariate logistic regression analysis, participants were categorized into low or high eHealth literacy groups based on mean scores for each domain. A threshold of 2.5 and above was used to define high eHealth literacy, indicating strong agreement with domain items. Backward stepwise logistic regression was performed for each of the 7 domains, with low eHealth literacy as the dependent variable. Independent variables included sex, age (treated as a continuous variable), education level, self-rated health (dichotomized), frequency of HRII acquisition, and perceived importance and usefulness of HRII acquisition (also dichotomized). Variance inflation factor and tolerance tests were conducted [39] to assess multicollinearity, revealing no indications of multicollinearity among the independent variables. Interactions between independent variables were also examined and not observed.

We reported the odds ratio and its corresponding 95% CI to assess the associations between eHealth literacy and various independent variables. The variance explained by the logistic regression models was evaluated using the Nagelkerke *r*^2^. Statistical significance was determined using a threshold of *P*<.05.

### Ethical Considerations

Ethical approval for this study was obtained from the regional ethical review board, Etikprövningsmyndigheten, (2019‐0341, 2014/179‐31), including a supplementary application for expanded data collection. All procedures and data management were conducted following the General Data Protection Regulation and ethical principles outlined in the Helsinki Declaration [40,41]. Informed consent was obtained from all study participants regarding data collection and the analysis of the data. The questionnaires were submitted entirely anonymously. No form of compensation was provided to the participants.

## Results

### Demographic Characteristics

A total of 172 questionnaires were collected. The sociodemographic characteristics of the sample are presented in Table 2.

### The eHealth Literacy Questionnaire

**Figure 1.**
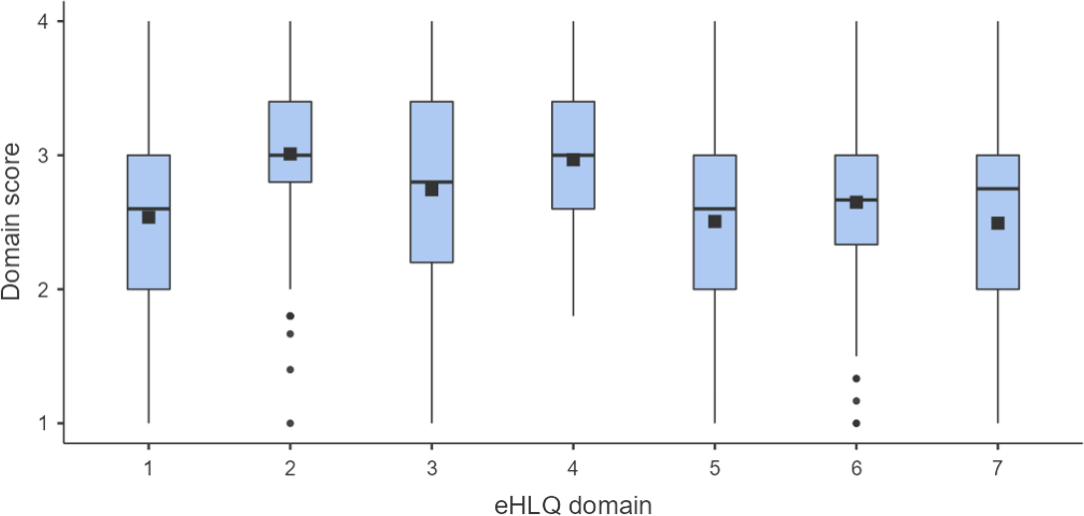
Distribution of eHLQ scores across different domains. eHLQ domains: (1) using technology to process health information, (2) understanding of health concepts and language, (3) ability to actively engage with digital services, (4) feeling safe and in control, (5) motivated to engage with digital services, (6) access to digital services that work, and (7) digital services that suit individual needs. eHLQ: eHealth Literacy Questionnaire.

As a group, participants rated highest on domains 2 and 4, indicating a strong perception of understanding and engagement with their health and feeling secure regarding the safety and confidentiality of eHealth services. Conversely, domains where participants rated lower included perceptions of whether eHealth services suited their personal needs (domain 7), motivation to use eHealth services (domain 5), and perceived ability to understand and use HRII (domain 1) (Figure 1 and Table 3).

**Table 3.** Mean and median scores of the 7 eHLQ^a^ domains.

Domain	Mean (SD)	95% CI	Median (IQR)
1. Using technology to process health information	2.54 (0.76)	2.42‐2.65	2.60 (2.0‐3.0)
2. Understanding of health concepts and language	3.01 (0.57)	2.92‐3.09	3.00 (2.8‐3.4)
3. Ability to actively engage with digital services	2.74 (0.85)	2.62‐2.87	2.80 (2.2‐3.4)
4. Feeling safe and in control	2.97 (0.56)	2.88‐3.05	3.00 (2.0‐3.0)
5. Motivated to engage with digital services	2.51 (0.72)	2.40‐2.61	2.60 (2.0‐3.0)
6. Access to digital services that work	2.65 (0.62)	2.56‐2.74	2.67 (2.3‐3.0)
7. Digital services that suit individual needs	2.49 (0.78)	2.38‐2.61	2.75 (2.0‐3.0)

a eHLQ: eHealth Literacy Questionnaire.

### Internet and HRII-Seeking Habits

The findings revealed that most participants accessed the internet daily; however, daily internet use was less frequent in the highest age group (>75 years), with approximately half reporting daily usage. Additionally, as age increased, there was a notable decline in the frequency of HRII searches. There were age-related differences in the primary choice for health-related inquiries, with a clear majority in the youngest age group primarily choosing the internet. In contrast, almost all in the oldest age group preferred health care providers.

Regarding HRII searches, the choice between Google, 1177.se, and other sources was distributed similarly across age groups although substantial missing values existed in the older age groups. The results also demonstrated that the perceived usefulness and importance of HRII were highest in the youngest age group, gradually diminishing with each successive age group (Table 4).

**Table 4.** Internet and HRII^a^ use and attitudes among the total sample and across different age groups, n (%).

		Total sample, n (%)	Age (years), n (%)			
			≤40	41‐60	61‐74	≥75
**Frequency of internet use**						
	Every day	141 (82)	44 (100)	40 (97.6)	35 (81.4)	22 (52.4)
	Less often or never	29 (16.9)	—^b^	1 (2.4)	8 (18.6)	20 (47.6)
	Missing	2 (1.1)	—	—	—	—
**Frequency of HRII acquisition**						
	Every week	27 (15.9)	10 (22.7)	8 (19.5)	5 (11.6)	3 (7.1)
	Every month	57 (33.5)	26 (59.1)	14 (34.1)	10 (23.3)	7 (16.7)
	Less often or never	86 (50.6)	8 (18.2)	19 (46.3)	27 (62.8)	31 (73.8)
	Missing	2 (1.2)	—	—	1 (2.3)	1 (2.4)
**The primary source of health information**						
	Health care	84 (48.8)	7 (15.9)	17 (41.5)	24 (55.8)	34 (81)
	Internet	58 (33.7)	27 (61.4)	17 (41.5)	12 (27.9)	2 (4.8)
	Other	30 (17.4)	10 (22.7)	7 (17)	7 (16.3)	6 (14.3)
**The primary source of HRII**						
	Google	70 (47.9)	23 (52.3)	17 (43.6)	19 (55.9)	10 (37)
	1177.se	68 (46.6)	20 (45.5)	22 (56.4)	13 (38.2)	13 (48.1)
	Other	8 (5.5)	1 (2.3)	—	2 (5.9)	4 (14.8)
	Missing	26 (15.1)	—	2 (4.9)	9 (20.9)	15 (35.7)
**Perceived HRII usefulness**						
	Not useful	28 (17.3)	—	2 (5)	8 (20)	16 (43.2)
	Unsure	32 (19.8)	6 (14)	7 (17.5)	9 (22.5)	10 (27)
	Useful	102 (63)	37 (86)	31 (77.5)	23 (57.5)	11 (29.7)
	Missing	10 (5.8)	1 (2.3)	1 (2.4)	3 (7)	5 (11.9)
**Perceived HRII importance**						
	Not important	27 (16.7)	0 (0)	5 (12.5)	5 (12.5)	16 (43.2)
	Unsure	15 (9.3)	2 (4.7)	—	8 (20.0)	5 (13.5)
	Important	120 (74.1)	41 (95.3)	35 (87.5)	27 (67.5)	16 (43.2)
	Missing	10 (5.8)	1 (2.3)	1 (2.4)	3 (7.0)	5 (11.9)

a HRII: health-related internet information.

b 0 participants responded.

### eHealth Literacy in Relation to Sociodemographic Factors and Internet Habits

Univariate analyses revealed that men had significantly lower mean values across all scales, except for those related to the ability to use digital technology and motivation for eHealth usage (domains 3 and 5), with mean differences ranging from 0.17 to 0.28 units (*P*<.01 to *P*=.04). Additionally, differences were observed among different age groups across all eHLQ domains, with significantly lower scores observed as age increased. The largest differences between the youngest (<40 years) and oldest (>75 years) age groups were observed in domain 1 (using technology to process health information) and domain 3 (ability to actively engage with digital services), with mean differences of 0.88 units (*P*<.001) and 1.25 units (*P*<.001), respectively. Individuals with lower levels of education exhibited lower eHealth literacy across all domains except domain 4 (feeling safe and in control), with the largest mean differences observed between those with primary and university education in domain 1 (using technology to process health information, 0.88 units, *P*<.001) and domain 3 (ability to actively engage with digital services, 1.25 units, *P*<.001). Similarly, individuals with low self-rated health demonstrated lower mean values across all domains except domain 4, with mean differences between those with poor or somewhat okay self-rated health and those with excellent self-rated health ranging from 0.34 to 0.63 units (*P*<.001 to *P*=.005). Individuals who perceived HRII as not useful rated significantly lower across all eHLQ domains, compared to those who perceived it as useful, with mean differences ranging from 0.32 to 1.57 units (*P*<.001 to *P*<.002). Similarly, those who perceived HRII as not important scored lower across all domains, compared to those who considered it important, with mean differences ranging from 0.34 to 1.45 units (*P*<.001 to *P*=.04) (Multimedia Appendix 1).

The final multivariate logistic regression analysis revealed associations between low eHealth literacy and higher age in domains related to finding, understanding, and using HRII (domain 1), using digital technology (domain 3), and accessing well-functioning eHealth services (domain 6). The perception that HRII was not useful was associated with lower eHealth literacy in domains related to individual capabilities (domains 1 and 2: understanding one’s health and using HRII), the interaction between the individual and eHealth services (domains 3‐5: ability and motivation to use digital technology and feelings of security), and characteristics of the eHealth system (domain 6: access to functional eHealth services).

Perceiving HRII as not important was associated with lower eHealth literacy in domains related to the use of HRII (domain 1), motivation to use eHealth (domain 5), and eHealth services that suited personal needs (domain 7). Poorer self-rated health was associated with lower perceived motivation to use eHealth services (domain 5) (Table 5). In the logistic regression analysis, other covariates included in the initial models, such as sex, education, and frequency of accessing HRII, showed no association with low eHealth literacy.

**Table 5.** Final multivariate logistic regression models and their associations for low eHealth literacy. Independent variables in the initial models included sex, age, education, self-rated health, frequency of HRII^a^ acquisition, and perceived importance and usefulness of HRII.

eHLQ^b^ domain and significant independent variables		OR^c^ (95%CI)	*P* value	*r* ^2^ ^d^
**1. Using technology to process health information**				0.51
	Higher age	1.02 (1.00‐1.05)	.03	
	HRII not useful	4.16 (1.64‐10.56)	.003	
	HRII not important	9.50 (2.72‐33.16)	<.001	
**2. Understanding of health concepts and language**				0.20
	HRII not useful	7.41 (2.76‐19.90)	<.001	
**3. Ability to actively engage with digital services**				0.53
	Higher age	1.05 (1.02‐1.08)	<.001	
	HRII not useful	12.32 (4.98‐30.49)	<.001	
**4. Feeling safe and in control**				0.08
	HRII not useful	3.12 (1.46‐6.70)	<.001	
**5. Motivated to engage with digital services**				0.43
	Poorer self-assessed health	2.64 (1.15‐6.01)	.02	
	HRII not useful	4.47 (1.81‐10.74)	<.001	
	HRII not important	6.72 (2.13‐21.20)	<.001	
**6. Access to digital services that work**				0.23
	Higher age	1.02 (1.00‐1.05)	.03	
	HRII not useful	3.85 (1.72‐8.64)	<.001	
**7. Digital services that suit individual needs**				0.18
	HRII not important	10.64 (3.12‐36.32)	<.001	

a HRII: health-related internet information.

b eHLQ: eHealth Literacy Questionnaire.

c OR: odds ratio.

d Using the Nagelkerke model.

## Discussion

### Principal Findings

The main finding from this study was that higher age and perceptions of HRII as not useful or important were the primary factors associated with lower eHealth literacy among our sample of primary health care visitors. Notably, the domains in which participants scored highest, specifically those related to understanding and engagement with health (domain 2) and a sense of security and control (domain 4), were not directly associated with using and interacting with the eHealth system.

The most challenging domains were 5 and 7, suggesting that many participants had low motivation to use eHealth services and perceived that these services did not meet their individual needs. Additionally, other domains where many participants indicated low eHealth literacy included the use of HRII (domain 1) and active engagement with digital services (domain 3). The findings regarding the domains rated highest and lowest align with studies conducted using the eHLQ in diverse international settings. For example, similar patterns have been observed among Spanish primary health care visitors [42], a representative Australian population [43], Taiwanese individuals with chronic diseases [35], Canadian cancer survivors [44], and a small-scale Swedish study focusing on parents of hospitalized children [30].

The observation that predominantly older participants exhibited lower eHealth literacy was expected and is consistent with prior research findings [28,34,42]. Domains of the eHLQ where low eHealth literacy was associated with higher age included searching, critically appraising, using HRII, using digital technology, and accessing well-functioning eHealth services. The reasons for these lower capabilities are often attributed to the limited integration of digital technology into their work and daily lives, resulting in less familiarity compared to younger generations. Older individuals may also prefer traditional health care management methods if these methods have proven satisfactory [28]. Moreover, older individuals are more likely to encounter physical obstacles that complicate the use of digital services, such as impaired vision, tremors, or cognitive limitations [18]. Among our participants, the older group primarily sought health-related inquiries from health care providers, unlike the younger group, which predominantly sought information on the internet. However, the eHealth challenges faced by older individuals can be addressed through initiatives such as increased exposure to the internet and eHealth services, as well as by adapting eHealth services to meet individual needs [28].

Among the participants in this study, a clear association was observed between low eHealth literacy and the perception that HRII was not useful and important—a perception most commonly held by older participants. Previous studies have also reported similar associations between low eHealth literacy and these attitudes toward HRII [29,45-47]. This association has been argued to be explained by the fact that favorable attitudes toward HRII may lead to frequent web-based searches and increased eHealth service use, which could contribute to improved eHealth literacy [48]. Conversely, it is reasonable to consider that the relationship could also be reversed; high eHealth literacy results in more positive attitudes, while low eHealth literacy leads to lower levels of eHealth usage and more negative attitudes [49].

We found that rating one’s health as poorer was associated with lower motivation to use eHealth. Similar relationships have been reported in previous studies [50-52]. Possible reasons for this association include the hypothesis that individuals with high eHealth literacy are more inclined to use digital resources for information about medical treatment and preventive actions to maintain good health [53].

These 7 eHLQ domains should not be regarded as isolated entities, as there may be a chain reaction wherein challenges in one domain could impact another. For instance, if eHealth services are perceived as not meeting individual needs (domain 7), it could decrease motivation to use these services (domain 5), subsequently leading to a reduced ability and inclination to seek HRII (domain 1). Furthermore, it is important not to view eHealth literacy and the factors influencing it as static entities; instead, they fluctuate and depend on the situation where an individual currently finds themselves [19,54].

To facilitate interventions aimed at improving eHealth literacy at both group and individual levels, health care professionals, eHealth developers, and health care authorities need to be aware of the areas within eHealth usage that pose the greatest challenges. Therefore, participants who assess their eHealth literacy as low are the target group for outreach and assistance in eHealth usage. Within our sample, many participants rated low on the perception that eHealth services meet their needs and the motivation to use eHealth. Notably, being motivated to use eHealth is often considered the foundation for overall eHealth service usage and is deemed more critical than having digital abilities [18,55]. However, it is conceivable that increased motivation arises only when eHealth services are perceived to meet personal needs or when individuals have learned to use eHealth services. Proposed measures to boost motivation to use eHealth could include health care professionals or eHealth educators presenting eHealth services in a relatable manner (used by “people like me”) and providing learning opportunities without pressure [56].

As health care professionals, facilitating the patient’s perception that eHealth services do not meet individual needs may involve offering person-centered recommendations for websites or applications tailored to the patient’s specific health condition, informational needs, and perceived capabilities. For eHealth developers and health care authorities, facilitative measures may involve engaging patients and health care professionals in the development of eHealth services and HRII, ensuring that HRII is universally designed, and enabling customization of eHealth services [16]. However, it is essential to acknowledge that there will always be a group of individuals who prefer and can only engage with traditional health care interactions and communication. Consequently, this option must be maintained alongside eHealth services [57].

### Limitations and Future Research

The data collection was conducted during the first year of the COVID-19 pandemic. This led to a reduced number of participating primary health care centers and limited time available for data collection, ultimately affecting the intended sample size. Using both web-based and paper-based questionnaires could have potentially increased the sample size. However, given that our study focused on internet use and health literacy competencies, we prioritized consistency and simplicity by administering only paper-based questionnaires.

Regarding eHealth literacy measurement, it is important to note that the eHLQ assesses people’s perceptions rather than their actual digital competencies. This implies that individuals may both underestimate and overestimate their skills [58].

Another limitation is that eHealth-literate individuals might be overrepresented in this study because they are more likely to participate than people who consider themselves to have poor eHealth literacy skills or negative attitudes toward eHealth service usage. This phenomenon is, however, complex to avoid. It is also important to note the low variance in some domains of the regression model, indicating the presence of factors influencing eHealth literacy that were not explored in this study. Based on existing research, these factors could include, for instance, a reluctance and fear of deviating from traditional physical contact with health care professionals, inadequate or negative experiences with digital usage, physical or psychological barriers to use (such as visual impairments, dementia, or tremors), or the absence of support from the surrounding environment regarding the use of digital tools [59,60]. However, we do not have data to confirm this; hence, it is an area that requires further investigation.

This study was conducted within a Swedish context, allowing readers to assess the transferability of the results to other settings based on the provided contextual descriptions. Notably, the sample included a higher proportion of women than the general population, and the average age of our sample was also higher than that of the community. Unfortunately, we lack data on the age distribution of primary care visitors in the county in relation to our sample. However, it is generally recognized that older individuals tend to use health care facilities more frequently. Furthermore, this study included only Swedish-speaking individuals, highlighting the necessity for future research to incorporate non-Swedish speakers who may face health care exclusion due to language barriers.

The focus of this study is on individuals who actively seek health care services. Future research should prioritize examining eHealth literacy among those not engaged in face-to-face care, as they may derive greater benefits from digital health options. Furthermore, it would be valuable to investigate whether individuals who prefer digital health services score differently than those who attend in-person consultations.

Future research should further explore low eHealth literacy through a qualitative lens, focusing on groups facing more significant challenges, such as older adults and those who perceive digital health tools as not useful or important. This approach would provide deeper insights into these individuals’ preferences for support and care, thereby facilitating the development of tailored interventions and the design of appropriate digital tools.

### Conclusions

This study has provided valuable insights into eHealth literacy among individuals within a Swedish primary health care setting by using a multidimensional approach highlighting specific domains where participants faced the greatest challenges. The most challenging areas identified included low motivation to use eHealth services and the perception that these services did not meet individual needs, as well as difficulties in using HRII and actively engaging with digital services. Results indicated that higher age and perceptions of HRII as not useful or important were the primary factors associated with lower eHealth literacy. Primary health care nurses and other professionals could play a crucial role in enhancing patients’ eHealth literacy by recommending personalized websites and eHealth services. This person-centered support can address patients’ attitudes and guide them toward services that better meet their needs, ultimately increasing their confidence and motivation to use eHealth. To ensure that eHealth services effectively align with users’ needs, developers and health care authorities need to involve patients and health care professionals in their development.

## Supplementary material

10.2196/63288 Multimedia Appendix 1 Associations between sociodemographic factors and eHealth Literacy Questionnaire (eHLQ) domains and between internet habits and eHLQ domains.

## Abbreviations

eHLF: eHealth Literacy Framework
eHLQ: eHealth Literacy Questionnaire
HRII: health-related internet information
OR: odds ratio
