# Supplementary material for: eHealth Literacy and Health-Related Internet Use Among Swedish Primary Health Care Visitors: Cross-Sectional Questionnaire Study
Source: JMIR Form Res. 2024 Dec 5;8:e63288. doi: 10.2196/63288 (PMC11637456; doi:10.2196/63288)
Supplement: Multimedia Appendix 1 [file formative-v8-e63288-s001.docx]

***Table.*** *Associations among sociodemographic factors and Internet habits and the seven eHLQ domains, mean (SD).*

| **eHLQ domain** | **1** | **2** | **3** | **4** | **5** | **6** | **7** |
| --- | --- | --- | --- | --- | --- | --- | --- |
| **Total sample** | 2.54 (0.76) | 3.01 (0.57) | 2.74 (0.85) | 2.97 (0.56) | 2.51 (0.72) | 2.65 (0.62) | 2.49 (0.78) |
| **Sex** |  |  |  |  |  |  |  |
| Female | 2.66 (0.72) | 3.08 (0.56) | 2.83 (0.79) | 3.06 (0.55) | 2.60 (0.68) | 2.75 (0.61) | 2.58 (0.75) |
| Male | 2.38 (0.79) | 2.91 (0.56) | 2.62 (0.90) | 2.85 (0.55) | 2.39 (0.75) | 2.52 (0.61) | 2.36 (0.79) |
| *P*-value | .02 | .04 | .13 | .02 | .053 | .01 | .04 |
| Effect size^dd^ | 0.37 | 0.29 | 0.25 | 0.38 | 0.30 | 0.38 | 0.29 |
| **Age groups** |  |  |  |  |  |  |  |
| ≤40^a^ | 3.01 (0.55)^c,d^ | 3.19 (0.58)^c^ | 3.40 (0.53)^b,c,d^ | 3.15 (0.59) | 2.90 (0.68)^c,d^ | 2.92 (0.57)^c,d^ | 2.95 (0.78)^c,d^ |
| 41-60^b^ | 2.69 (0.70)^d^ | 3.13 (0.51)^c^ | 3.01 (0.70)^c,d^ | 3.06 (0.58) | 2.66 (0.60)^d^ | 2.77 (0.55) | 2.63 (0.61)^d^ |
| 61-74^c^ | 2.34 (0.68)^a^ | 2.87 (0.44)^a,b^ | 2.44 (0.70)^a,b^ | 2.85 (0.41) | 2.34 (0.57)^a^ | 2.54 (0.50)^a^ | 2.26 (0.59)^a^ |
| ≥75^d^ | 2.13 (0.79)^a,b^ | 2.88 (0.66) | 2.15 (0.78)^a,b^ | 2.81 (0.57) | 2.15 (0.77)^a,b^ | 2.41 (0.70)^a^ | 2.16 (0.81)^a^ |
| *P*-value | <.001 | .002 | <.001 | .03 | <.001 | <.001 | <.001 |
| Effect size^ee^ | 0.20 | 0.09 | 0.37 | 0.06 | 0.16 | 0.12 | 0.18 |
| **Education** |  |  |  |  |  |  |  |
| Elementary school or less^e^ | 2.13 (0.93)^g^ | 2.83 (0.53)^g^ | 2.09 (0.90)^f,g^ | 2.80 (0.52) | 2.22 (0.87)^g^ | 2.36 (0.73)^g^ | 2.25 (0.93) |
| Secondary school or vocational^f^ | 2.45 (0.74)^g^ | 2.84 (0.59)^g^ | 2.64 (0.84)^e,g^ | 2.91 (0.56) | 2.36 (0.69)^g^ | 2.61 (0.62) | 2.42 (0.73) |
| University^g^ | 2.78 (0.63)^e,f^ | 3.25 (0.47)^e,f^ | 3.09 (0.65)^e,f^ | 3.09 (0.55) | 2.76 (0.62)^e,f^ | 2.80 (0.54)^e^ | 2.66 (0.74) |
| *P*-value | .002 | <.001 | <.001 | .08 | <.001 | .04 | .04 |
| Effect size^ee^ | 0.14 | 0.08 | 0.15 | 0.03 | 0.09 | 0.04 | 0.04 |
| **Self-reported health** |  |  |  |  |  |  |  |
| Poor or somewhat ok^h^ | 2.24 (0.72)^I,j^ | 2.85 (0.62)^j^ | 2.47 (0.82)^j^ | 2.87 (0.54) | 2.20 (0.69)^i,j^ | 2.46 (0.58)^i,j^ | 2.23 (0.72)^I,j^ |
| Good^i^ | 2.53 (0.81)^h^ | 2.99 (0.54) | 2.75 (0.90) | 3.00 (0.55) | 2.51 (0.75)^h^ | 2.69 (0.66)^h^ | 2.55 (0.83)^h^ |
| Very good/excellent^j^ | 2.86 (0.62)^h^ | 3.21 (0.50)^h^ | 3.02 (0.74)^h^ | 3.05 (0.56) | 2.83 (0.58)^h^ | 2.80 (0.58)^h^ | 2.73 (0.70)^h^ |
| *P*-value | <.001 | .003 | .003 | .19 | <.001 | .005 | <.001 |
| Effect size^ee^ | 0.11 | 0.07 | 0.07 | 0.02 | 0.12 | 0.06 | 0.08 |
| **Frequency of Internet use** |  |  |  |  |  |  |  |
| Every day^aa^ | 2.70^bb,cc^ | 3.05 | 2.94^bb,cc^ | 2.99 | 2.64^bb,cc^ | 2.73^cc^ | 2.60^bb,cc^ |
| A couple of times/week or month^bb^ | 1.84^aa^ | 2.76 | 1.89^aa^ | 2.89 | 2.16^aa^ | 2.35 | 1.95^aa^ |
| Less often or never^cc^ | 1.63^aa^ | 2.84 | 1.67^aa^ | 2.81 | 1.69^aa^ | 2.17^aa^ | 1.85^aa^ |
| *P*-value | <.001 | .09 | <.001 | .38 | <.001 | <.001 | <.001 |
| Effect size^ee^ | 0.20 | 0.03 | 0.25 | 0.02 | 0.16 | 0.09 | 0.11 |
| **eHLQ domain** | **1** | **2** | **3** | **4** | **5** | **6** | **7** |
| **Frequency of HRII acquisition** |  |  |  |  |  |  |  |
| Every week^k^ | 3.01 (0.55)^m^ | 3.27 (0.50)^m^ | 3.13 (0.67)^m^ | 3.08(0.53) | 2.96 (0.62)^m^ | 2.88 (0.58)^m^ | 2.84 (0.79)^m^ |
| Every month^l^ | 2.85 (0.56)^m^ | 3.12 (0.54)^m^ | 3.13( 0.61)^m^ | 3.00(0.54) | 2.71 (0.64)^m^ | 2.74 (0.53) | 2.64 (0.72)^m^ |
| Less often or never^m^ | 2.17 (0.77)^k,l^ | 2.85 (0.57)^k,l^ | 2.36 (0.88)^k,l^ | 2.92(0.57) | 2.22 (0.69)^k,j^ | 2.51 (0.66)^k^ | 2.28 (0.76)^k,j^ |
| *P*-value | <.001 | <.001 | <.001 | .36 | <.001 | .012 | < .001 |
| Effect size^ee^ | 0.23 | 0.09 | 0.20 | 0.01 | 0.17 | 0.05 | 0.08 |
| **Primary source of health information** |  |  |  |  |  |  |  |
| Healthcare^n^ | 2.07 (0.71)^o,p^ | 2.88 (0.61)^o,p^ | 2.27 (0.84)^o,p^ | 2.93 (0.60) | 2.12 (0.66)^o,p^ | 2.47 (0.67)^o,p^ | 2.23 (0.75)^o,p^ |
| The Internet^o^ | 2.94 (0.52)^n^ | 3.10 (0.54)^n^ | 3.21 (0.61)^n^ | 3.00 (0.53) | 2.81 (0.60)^n^ | 2.82 (0.57)^n^ | 2.72 (0.78)^n^ |
| Other^p^ | 3.05 (0.45)^n^ | 3.19 (0.37)^n^ | 3.13 (0.51)^n^ | 3.00 (0.50) | 2.99 (0.48)^n^ | 2.81 (0.43)^n^ | 2.77 (0.59)^n^ |
| *P*-value | <.001 | .01 | <.001 | .67 | <.001 | .003 | <.001 |
| Effect size^ee^ | 0.37 | 0.06 | 0.30 | 0.00 | 0.30 | 0.07 | 0.11 |
| **Primary source of HRII** |  |  |  |  |  |  |  |
| Google^q^ | 2.82 (0.61)^s^ | 3.06 (0.53) | 2.98 (0.72) | 2.90 (0.52) | 2.64 (0.65) | 2.70 (0.54) | 2.54 (0.76) |
| 1177^r^ | 2.60 (0.71) | 3.06 (0.58) | 2.88 (0.77) | 3.09 (0.54) | 2.62 (0.66) | 2.77 (0.55) | 2.68 (0.65) |
| Other^s^ | 1.77 (0.87)^q^ | 2.99 (0.81) | 1.99 (1.23) | 2.97 (0.64) | 1.91(0.99) | 2.23 (1.03) | 2.41 (1.20) |
| *P*-value | .004 | .95 | .06 | .18 | .08 | .19 | .58 |
| Effect size^ee^ | 0.08 | 0.00 | 0.04 | 0.02 | 0.04 | 0.02 | 0.01 |
| **HRII usefulness** |  |  |  |  |  |  |  |
| Not useful^t^ | 1.55 (0.54)^u,v^ | 2.58 (0.65)^u,v^ | 1.59 (0.61)^u,v^ | 2.77 (0.59)^v^ | 1.66 (0.59)^u,v^ | 2.00 (0.59)^u,v^ | 1.74 (0.81)^u,v^ |
| Unsure^u^ | 2.28 (0.54)^t,v^ | 2.76 (0.48)^t,v^ | 2.56 (3.16)^t,v^ | 2.77 (0.50)^v^ | 2.23 (0.55)^t,v^ | 2.51 (0.37)^t,v^ | 2.29 (0.54)^t,v^ |
| Useful^v^ | 2.92 (0.55)^t,u^ | 3.23 (0.45)^t,u^ | 3.16 (0.58)^t,u^ | 3.09 (0.53)^t,u^ | 2.85 (0.55)^t,u^ | 2.87 (0.52)^t,u^ | 2.78 (0.67)^t,u^ |
| *P*-value  Effect size^ee^ | <.001  0.44 | <.001  0.25 | <.001 0.42 | <.002 0.08 | <.001 0.39 | <.001 0.27 | <.001 0.26 |
| **HRII importance** |  |  |  |  |  |  |  |
| Not important^x^ | 1.52 (0.61)^y,z^ | 2.59 (0.71)^z^ | 1.64 (0.73)^y,z^ | 2.69 (0.61)^z^ | 1.60 (0.61)^y,z^ | 2.05 (0.64)^y,z^ | 1.68 (0.66)^y,z^ |
| Unsure^y^ | 2.12 (0.30)^x,z^ | 2.89 (0.50) | 2.40 (0.44)^x,z^ | 3.01 (0.38) | 2.24 (0.31)^x,z^ | 2.54 (0.36) | 2.35 (0.64) |
| Important^z^ | 2.85 (0.57)^x,y^ | 3.14 (0.48)^x^ | 3.07 (0.66)^x,y^ | 3.03 (0.54)^x^ | 2.77 (0.59)^x,y^ | 2.79 (0.53)^x^ | 2.71 (0.69)^x^ |
| *P-*value  Effect size^ee^ | <.001  0.40 | <.001  0.12 | <.001 0.35 | .04 0.06 | <.001 0.33 | <.001 0.18 | <.001 0.24 |

a-z and aa-cc represents a P <0.05 difference between groups in post hoc test
dd=Cohen´s D
ee=Epsilon square (ε2)
HRII=Health-related Internet information
eHLQ domains:

1. Using technology to process health information

2. Understanding of health concepts and language

3. Ability to actively engage with digital services

4. Feeling safe and in control

5. Motivated to engage with digital services

6. Access to digital services that work

7. Digital services that suit individual needs
